# Protein nanofibril design via manipulation of hydrogen bonds

**DOI:** 10.1038/s42004-021-00494-2

**Published:** 2021-05-11

**Authors:** Nidhi Aggarwal, Dror Eliaz, Hagai Cohen, Irit Rosenhek-Goldian, Sidney R. Cohen, Anna Kozell, Thomas O. Mason, Ulyana Shimanovich

**Affiliations:** 1 Department of Molecular Chemistry and Materials Science, Weizmann Institute of Science, Rehovot, Israel; 2 Department of Chemical Research Support, Weizmann Institute of Science, Rehovot, Israel

**Keywords:** Self-assembly, Biomaterials - proteins

## Abstract

The process of amyloid nanofibril formation has broad implications including the generation of the strongest natural materials, namely silk fibers, and their major contribution to the progression of many degenerative diseases. The key question that remains unanswered is whether the amyloidogenic nature, which includes the characteristic H-bonded β-sheet structure and physical characteristics of protein assemblies, can be modified via controlled intervention of the molecular interactions. Here we show that tailored changes in molecular interactions, specifically in the H-bonded network, do not affect the nature of amyloidogenic fibrillation, and even have minimal effect on the initial nucleation events of self-assembly. However, they do trigger changes in networks at a higher hierarchical level, namely enhanced 2D packaging which is rationalized by the 3D hierarchy of β-sheet assembly, leading to variations in fibril morphology, structural composition and, remarkably, nanomechanical properties. These results pave the way to a better understanding of the role of molecular interactions in sculpting the structural and physical properties of protein supramolecular constructs.

## Abstract (web summary)

How the structure of fibril-forming peptides influences the material and structural characteristics of their resultant assemblies remains an open question. Here, aliphatic to aromatic amino acid substitution in such peptides is shown to have little effect on the tendency to form amyloid-like fibrils, but instead alters the pathway by which they form and the mechanical properties of peptide supramolecular assemblies.

## Introduction

Protein self-assembly, in particular fibrillar type of self-assembly, is driven by non-covalent molecular interactions^1^. This type of self-assembly is linked to two opposing biological roles: (1) aberrant self-assembly, accompanied by the formation of amyloid fibrils^2,3^, associated with the development of multiple disorders, including neurodegenerative diseases such as Alzheimer’s and Parkinson’s; (2) and functional self-assembly, such as the formation of mechanically strong and, at the same time, elastic fibers (also called functional amyloids) like silk^4,5^. Interestingly, several studies revealed the interconnection and the critical role of the mechanical behavior of amyloid fibrils in expression and propagation of neurodegenerative disorders^6^. At the same time, functional amyloid nanostructures, due to their unique mechanical properties and biocompatibility, are increasingly viewed as promising building blocks for new biomaterials design and construction. Thus, understanding self-assembly pathway and processes by which the material properties of amyloids are shaped is highly important for both potential disease treatments and materials design^7–10^.

Despite variations in biological role and primary protein/peptide sequences in amyloid fibers, they are all share similar molecular organization. The amyloid supramolecular structures are generally characterized by β-strands oriented perpendicularly to the fibril axis, and connected through a dense hydrogen-bonded (H-bond) network^11^, which results in continuously extended supramolecular β-sheets. Natural proteins that contain such sequences are likely to be problematic for living organisms, due to their potential to aggregate into toxic structures. In particular, over the last few decades, several studies performed by different research groups around the world, have shown that sequences rich in A and/or G and /or AG repetitive motifs, in diverse groups of proteins, lead to formation of amyloid fibrils. For example, such sequences are known to increase risk of Huntington’s and mad cow diseases^12–14^. The mutations in which natively occurring amino acids replaced with GA are known to induce/trigger aggregation of α-synuclein (Parkinson’s), Aβ (Alzheimer’s), as well as to contribute to the development of many other diseases associated with amyloidogenic fibrillation of proteins and peptides. Interestingly, repetitive AG motifs are also found in functional amyloids, which are defined as protein/peptide fibrils having structural similarity with pathological constructs, but utilized by organisms in functional roles. Examples include eggshell chorion in silk morph, fibroin in silkworms, spidroin in spiders, Pmel17 which plays a central role in melanin-a polymerization in humans, and many more^15–17^. Furthermore, the presence of hydrophobic amino acids in amyloidogenic motifs are also known to induce fibrillation in proteins and peptides. Non-covalent molecular interactions, specifically hydrophobic, π–π stacking and H-bonds, can stabilize amyloidogenic structure in general. However, the “typical” amyloid structure does not primarily rely on side-chain interactions, but rather on universal physico-chemical characteristics of the protein/peptide backbone, which are contributing to the natural propensity for H-bond formation in the backbone^11^. Nonetheless, H-bonds are known to be a driving force for amyloidogenic aggregation^18^, while π-stacking interactions^19–23^ often accelerate the process of fibril formation by providing geometrical restrictions that promote directionality and orientation of the growing fibril. Thus, for example, evidence of the involvement of aromatic interactions in amyloid fibril formation has been reported for an amyloid-forming peptide Aβ, associated with Alzheimer’s disease, whose core sequence is KLV**FF**. Aβ fibrillation is initiated by mutual interactions between the hydrophobic, diphenylalanine (FF) motifs. As a consequence of the fibrillation process, the FFs are buried in the fibril core^24^. The fibril growth propagation is driven by the formation of an H-bonded network between amide groups (C=O and NH) of neighboring peptides. Opposite the above described Aβ fibrillation pathway, the self-assembly of a phenylalanine dipeptide, a peptide containing only FF residues, is triggered by H-bonds formation and is further propagated via π-π stacking interactions that delocalize F residues at the interface of peptide supramolecular assemblies^25^. Another example is viral capsid assembly, in which a large number (from 60 to 1000s) of protein subunits assemble into complete, reproducible structures under a variety of conditions while avoiding kinetic and thermodynamic traps^26,27^. Thus, all cases of the fibrillar self-assembly involve interactions between aromatic residues and the formation of an extensive H-bonded network. However, it still remains unclear how and why these relatively similar processes result in the formation of fibrils distinct in their material properties, and, in particular, their mechanical strength. Aβ peptide fibrils display an elastic modulus of 2–5 GPa^28,29^, while the Young modulus of FF assemblies ranges between 20 and 30 GPa^25^.

In this work, we have investigated how the molecular interactions, in particular H-bonds and interactions between aromatic residues, shape the physical properties of self-assembled amyloid protein constructs. To this end, we have introduced steric constraints into a fibrillar amyloidogenic peptide model by substituting G with aromatic (F, Y, or W) amino acids in the core sequence GAGAGSGAGAGSGAGAGSGAG. Thus, two types of imposed changes in molecular interactions were introduced: (1) the introduction of aromatic residues in the core of the fibril backbone, such that steric constraints would interfere with the H-bonded network formation; (2) delocalization of aromatic residues at the fibril interface, to promote better overlap between peptide backbones and facilitate continuous H-bonded network formation, and/or limit fibril elongation due to the geometrical constraints created at fibril termini. The aromatic amino acid substitutes differ in their relative hydrophobicity, polarity, and ability to form H-bonds. Therefore, the chosen core sequence of the peptide is relevant to both functional and aberrant self-assembly manifestations^30^. On the one hand, the chosen sequence mimics the core sequence of the functional silk fibroin^31–33^. On the other hand, the chosen sequence also mimics the disease-associated fibrillar aggregation behavior of disordered proteins and peptides found in nature^30^.

Our study shows that deviations in the H-bonded networks of the fibrillating peptides do not necessarily interfere with their natural propensity toward amyloid formation; however, they do severely affect the folds, which define the fibrillation pathway in the mature state of the fibrils, which is directly reflected in changes in nanomechanical properties of fibrillar assemblies. This work thus demonstrates that controlled intervention in the molecular interactions and H-bonded network formation pathway enables the manipulation of the end-point structure and the mechanical properties of fibrillar proteins. This finding, therefore, not only sheds new light on the role of H-bonds in aberrant protein aggregation—it also opens the way to utilizing the basic principles of controlled intervention into molecular interactions and H-bonded network formation in amyloidogenic protein fibrils in rational materials design.

## Results and discussion

**Fig. 1:**
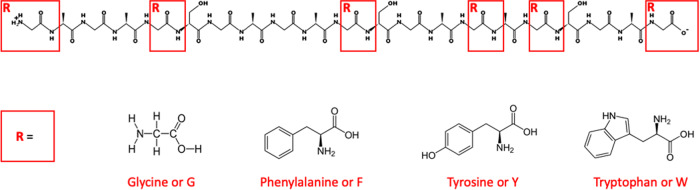
Schematics for chemical structure of peptide model and glycine to aromatic amino acid substitution. General chemical structure of peptides with a core GAGAGSGAGAGSGAGAGSGAG sequence. “R” corresponds to the amino acid substitution, represented by phenylalanine (F), tyrosine (Y), or tryptophan (W).

**Fig. 2:**
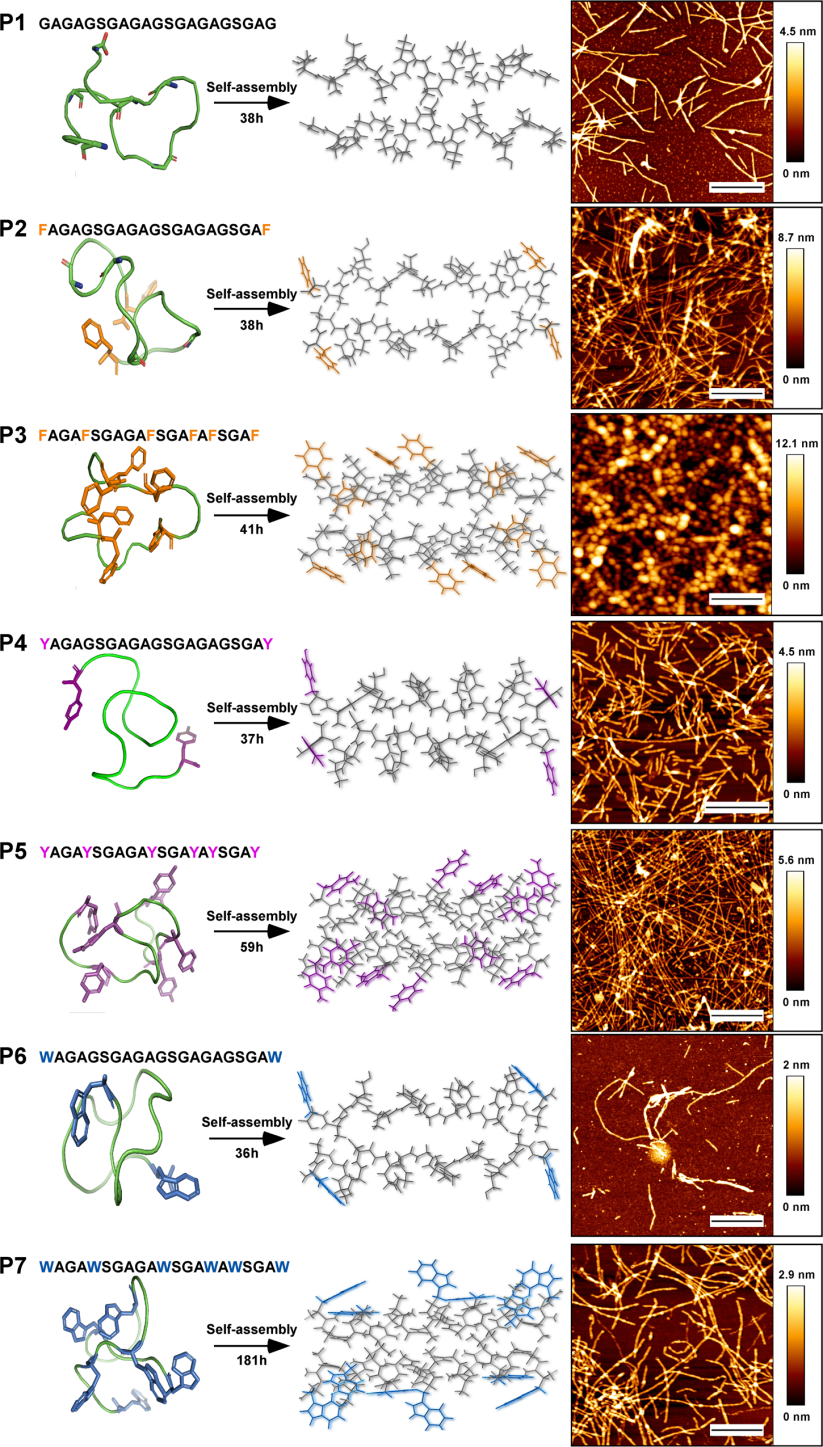
P1-P7 peptides’ supramolecular fibrillar self-assemblies. (P1) in which G is substituted with F in (P2), Y in (P4), or W in (P6) aromatic amino acids (with 10% substitution) and when the fraction of F (P3), Y (P5), and W (P7) was increased to 30%. The peptide structure (left column) was predicted using PEP-FOLD3 software (shown in ribbon and stick formats)^65^. The interaction between two peptide molecules schematically shown in the central column. From our experimental results, we observed that in all the cases the hydrophobic residues face an external part. The morphology of the self-assembled peptides, determined by AFM (the right panel), indicates the existence of polymorphism. The scale bars for AFM images are 500 nm.

To reveal how the changes in amyloidogenic motif sequences give rise to fluctuations in protein-protein interactions and contribute to the related material properties of protein fibrils, we explored a peptide design approach that enables artificial interference in molecular interactions and, consequently, in H-bond network formation, in a highly controlled manner. To this end, we selected a representative polypeptide chain sequence (GAGAGSGAGAGSGAGAGSGAG, shown in Fig. 1) with an intrinsically disordered structure as predicted by PEP-FOLD 3 software (see the methods section) and depicted in Fig. 2 (left column) and a high propensity for amyloidogenic fibrillar aggregation. Due to the highly hydrophobic nature of the designed peptides, they were solubilized in polar, hydrophobic dimethyl sulfoxide (DMSO) solvent. One might question the relevance of DMSO to intra- or extracellular conditions^34^, but based on multiple studies, it has been shown that DMSO does not abolish the biological activity of the cell in whole, as well as specific cellular components, including amyloidogenic aggregation-prone proteins^23,35^. Moreover, the liquid phases in the cytoplasm and in extracellular vicinity (close to the receptor sites), are characterized by high viscosity (higher than water and equivalent to DMSO or other high viscosity solvents) suggesting that DMSO is more realistic for modeling these environments and studying the behavior of highly hydrophobic biological compounds^23,35,36^. Furthermore, the high aggregation propensity and low solubility of the amyloidogenic proteins and peptides also result in inherent difficulties in the experimental handling and investigation by biophysical techniques and in cell cultures^37–40^. These technical difficulties significantly hamper the research into functional and pathological amyloids, and therefore, a variety of protocols to improve their solubilization have been developed and are available in the literature. In such peptide purification procedures, the first highly important step is the solubilization of peptides in organic solvents such as DMSO, ethanol, methanol, dichloromethane, trifluoroacetic acid, hexafluoroisopropanol, and their mixtures. Notably, DMSO is known as an aggregation inhibitor that stabilizes either natively folded states of peptide/protein monomers or misfolded disordered states. We recognize that indeed, solubilization of peptides in DMSO most probably affects the secondary structure of peptide amyloid intermediates (as has been explained number of literature reports), however, there is no evidence of changes in end-point amyloid fibril structure induced by DMSO. Thus, due to the highly hydrophobic nature of our peptide models, we have chosen DMSO as an optimal solvent that presumably causes no changes in final structure of amyloid assemblies.

We systematically substituted the amino acid Glycine (G) for one of the three different types of aromatic amino acids (see Fig. 1): Phenylalanine (F), Tyrosine (Y), and Tryptophan (W). Phenylalanine is a highly hydrophobic, non-polar amino acid with an aromatic ring that can both promote amyloidogenic fibrillation, by creating a physical proximity between two monomers through π–π stacking interactions^41^, or interfere with monomer–monomer interactions, by introducing steric hindrance^42^. Tyrosine, on the other hand, is an amphipathic, polar, uncharged amino acid with an OH group on an aromatic ring; it is characteristically a strong H-donor capable of promoting H-bond formation. Finally, Tryptophan is a non-polar aromatic amino acid that can both form an H-bond through its NH group on the indole ring and create a steric effect.

### Morphology of fibrillar assemblies

Atomic force microscopy (AFM) and transmission electron microscopy were applied to assess the dependence of the fibril surface morphology on the presence of the three different types of aromatic amino acids (F, Y, and W; Fig. 2 and Supplementary Fig. S1). Well-ordered and elongated fibrillar structures with lengths ranging from 100 nm to 1.5 µm were formed by peptides with the core GAGAGSGAGAGSGAGAGSGAG sequence (Fig. 2, P1), i.e., with no aromatic residues. The substitution of G with F (FAGAGSGAGAGSGAGAGSGAF), only at the two termini, led to the formation of relatively longer nanofibrils, at the micron scale, whereas the substitution of G with Y (YAGAGSGAGAGSGAGAGSGAY) led to the formation of shorter nanofibrils, 100−500 nm in length (Fig. 2, P2 and P4, respectively; the length distribution is presented in Supplementary Fig. S2a). The increase in the nanofibril length formed from P2 peptide indicates that the presence of F rings at two ends of the peptide termini limits the structural flexibility; therefore, allowing better overlap between the peptide backbones. The reduction in length of P4 fibrils most likely originates from the steric H-donor competitor (the OH group in Y’s aromatic residues) generating localized intervention in H-bond formation and limiting the fibril elongation process. This observation was reconfirmed by measuring the chemical kinetics (the results are shown in Supplementary Fig. 5a and b and discussed in detail in the “Kinetics” section).

Substituting G with W (WAGAGSGAGAGSGAGAGSGAW) results in the co-existence of two types of morphologies (Fig. 2, P6): spherical and fibrillar (50−150 nm). Since no π-π stacking interactions were detected (based on IR spectroscopy analysis) for the spherical assemblies, we can explain this observation by the formation of an H-bonded network between the peptide backbones, where W residues generate a steric interference that limits the fibrillation process in general (Supplementary Fig. S3). To determine the impact of the aromatic amino acid fraction (%) and arrangement on the resulting morphologies, we increased the fraction of each of the aromatic residues in the peptide sequence to ~30% (i.e., the number of aromatic amino acids per sequence was increased from two to six). The 30% G-to-F substitution severely affected the topology of the fibrils, leading to the formation of fibril-like structures assembled from beads of 20−80 nm in diameter (Fig. 2, P3) and a small fraction of fibrillar assemblies. The spherical assemblies with a diameter of 40−60 nm accounted for >50% of the population (Supplementary Fig. S2b). This observation is consistent with previous literature reports on spherical assembly formation, with characteristic diameters of 10–100 nm, in F-containing compounds, including diphenylalanine and diphenyl glycine^43^.

These spherical structures are generally considered as a transient and unstable state in amyloidogenic self-assembly pathway that further tend to reassemble into fibrils, and indicate that F residues indeed create steric constraints, but also increasing the role of the interaction with the solvent, DMSO, thus making the spherical type of assembly energetically more favorable^43–45^.

In contrast, we found that peptides featuring a 30% G-to-Y or G-to-W substitution form continuous fibrils, whose formation became possible via the OH and NH groups of the Y and W residues, respectively. The length for the majority of fibrils with 30% G-to-Y substitution was in the micron range, whereas the 30% G-to-W substituted ones had an equal distribution of fibrils with lengths of 100−500 nm, 500−900 nm, and >900 nm (Fig. 2 and Supplementary Fig. S2a).

### Kinetics of aggregation

Next, we investigated the chemical kinetics of aggregating peptides by using a standard Thioflavin T dye (ThT) binding assay^46,47^. Traditionally, the process of amyloidogenic self-assembly is defined as a two-step condensation process, namely, nucleation, a rate-limiting step characterized by high Gibbs free energy^48^, followed by fibril growth, forming fibers rich in cross β-structures^48–50^. We monitored the aggregation process, as well as the specific molecular events (i.e., nucleation and elongation), by following the changes in the emission spectra of ThT dye, which undergoes a red-shifted emission upon binding to amyloid structures. Our results show that the peptides with a low percentage of aromatic residues content (P2, P4, and P6) nucleated within 36−38 h, similar to P1. In contrast, those with a higher fraction of aromatic amino acid content (P3, P5, and P7) took 41–181 h to nucleate (Supplementary Fig. S4a and b). The longest nucleation time, 181 h, was recorded for the peptide with 30% W content. These results point to differences in the thermodynamic stabilities^2^ of the peptides, which are reflected in variations in the kinetics of the aggregation process, specifically in the nucleation step. Thus, for example, the greater solubility of P7 results in higher metastability^51^. The high metastability impedes the rearrangement/misfolding process (to form an initial/first interaction between the backbone atoms and/or backbone atoms and side-chain atoms), thus impeding the nucleation process. Moreover, analysis of the elongation rates (Supplementary Fig. S4b) of the fibrillation kinetics (Supplementary Fig. S4a) revealed a pronounced difference between the peptides. Thus, the highest elongation rate was observed for P5, which favors peptide–peptide molecular interaction and H-bonded network formation due to the high content of OH-rich Y. Reduced Y content, e.g., was reflected in slower elongation rates (for P4). Interestingly, the presence of an aromatic amino acid F, in P2 and P3, also resulted in a relative increase in elongation rates, compared to P1, P6, and P7. An overall analysis of the chemical kinetics of peptide self-assembly suggests that geometrical constraints, created by the presence of aromatic residues in peptide sequences, indeed interferes in the process of primary nuclei formation (Supplementary Fig. S4c), leading to longer nucleation times for peptides with higher fraction of aromatic residues. However, as soon as primary nuclei formed, these geometrical restrictions promote directionality (via H-bond formation) and collective orientation of the growing fibrillar structures (Supplementary Fig. S4d). Thus, the overall fibrillation process is balanced by the three main characteristics of peptide–peptide interaction: solvation, steric effect, and the capability to form bonds (H-bonds). The solvation and consequent peptide metastability would determine the rate of nucleation, and H-bond formation. The steric effect will affect the further growth of amyloidogenic fibrils- i.e. elongation process.

### Secondary structure

To gain further insights into the structural organization of peptide morphologies, we performed a Fourier transform infra-red spectroscopy (FT-IR) analysis, in which we followed the changes in the vibration bands corresponding to amide I, which highlight variations in intermolecular β-sheet and antiparallel amyloid β-sheet-rich fragments. Generally, the vibrational spectra of proteins/peptides are characterized by two major bands, namely, amide I (1600–1700 cm^−1^) and amide II (1480–1600 cm^−1^), which correspond to C=O and NH bend/CH stretching, respectively^52^ and, to a lesser extent, by amide A bands (>3000 cm^−1^). The amide I region is used to characterize the secondary structure of proteins/peptides, e.g., their intermolecular β-sheet (1610–1625 cm^−1^), native β-sheet (1625–1635 cm^−1^), random coil/ α-helix (1635–1665 cm^−1^), β-turn (1665–1690 cm^−1^), and antiparallel amyloid β-sheets (1690–1705 cm^−1^)^53^. In our study, we followed the changes in the FT-IR spectra corresponding to an intermolecular β-sheet and an antiparallel amyloid β-sheet.

We found that the peptide assemblies exhibit variations in their secondary structure (Supplementary Fig. S5). More specifically, the assemblies of all seven peptides showed the presence of aggregative β-sheet content with characteristic peaks at 1623 and 1695 cm^−1^; this is in agreement with reports for antiparallel β-sheet organization. Interestingly, in addition to characteristic β-sheet bands, in P1 we observed a C=O stretching band at 1730 cm^−1^, corresponding to the non-H-bonded carboxylic group, amide II CN, and a NH bending band at 1546 cm^−1^, indicative of non-H-bonded NH groups as well as an amide A NH band at a lower energy (<3300 cm^−1^), attributed to an H-bonded NH group with a small shoulder at ~3450 cm^−1^ that corresponds to the presence of non-bonded OH groups (naturally present in S residues). This observation indicates that not all the carboxyl and amine groups are involved in H-bonded network formation. The substitution of G with F, in P2 and P3, resulted in marginal changes in the secondary structure, as reflected in the appearance of a “shoulder” (1644–1682 cm^−1^) in aggregated β-sheet content, is attributed to the relative increase in the random coil/α-helical fraction. We observed the disappearance of the 1546 and 1730 cm^−1^ bands (non-H-bonded NH and C=O groups) and the appearance of a new band at 1518 cm^-1^ of amide II vibration, confirming that peptide adopts a β-turn type of structure. In addition, a small band at 3055 cm^−1^, attributed to the π–π stacking interactions between aromatic rings, has also been observed. This observation is in good agreement with kinetics measurements. It also highlights the role of the steric effect, created by F residues, inducing the proximity between peptide molecules that propagate via the formation of an H-bonded network. Whereas in the case of P2 the small steric hindrance promotes the formation of elongated structures, in P3 the more pronounced hindrance stabilizes an intermediate form of assembly, namely, spherical structures, with preservation of H-bonds. The tyrosine residues (Y), present in P4 and P5, induce β-sheet formation, through their OH group, which acts as a H donor, and also preserve native structural content consisting of random coil and α-helix. Thus, we observed the appearance of a 1730 cm^−1^ band (non-H-bonded C=O groups) and the disappearance of ~3450 cm^−1^ shoulder, which corresponds to the presence of non-bonded OH groups. Such a combination enables the formation of fibrils with the longest persistent length while preserving dominant disordered random coil/α-helical conformation. Such a phenomenon of a structural organization, driven by H-bond formation via Y residues, has been utilized by nature to form long fibrils with unusual mechanical properties, including superelasticity and contractibility^54–56^. Finally, the structural analysis of P6 and P7 has shown that the substitution of amino acid G with aromatic W induces aggregative β-sheet formation, and the preservation of disordered random coil/α-helical content (an indicative shoulder at 1640–1685 cm^−1^).

### Hydrogen bond characterization

We used X-ray photoelectron spectroscopy (XPS) to gain insights into the molecular structure and the formation of an H-bonded network within the peptide P1–P7 self-assemblies. In this approach, H-bonded network evaluation via XPS analysis (for which the only element that cannot be detected is hydrogen) is based on changes in the binding energies of neighboring atoms that were found to undergo charge transfer upon H-bond formation. We first inspected the composition of the various self-assembled structures (P1–P7) by quantifying the main resolvable features. The results are summarized in Supplementary Table S1 and Supplementary Note 1 for three major components in the C 1s line and the total signal of the O 1s and the N 1s lines. In general, we found good agreement with the theoretical stoichiometry for the amide groups, chosen as a reference for the XPS analysis.

**Fig. 3:**
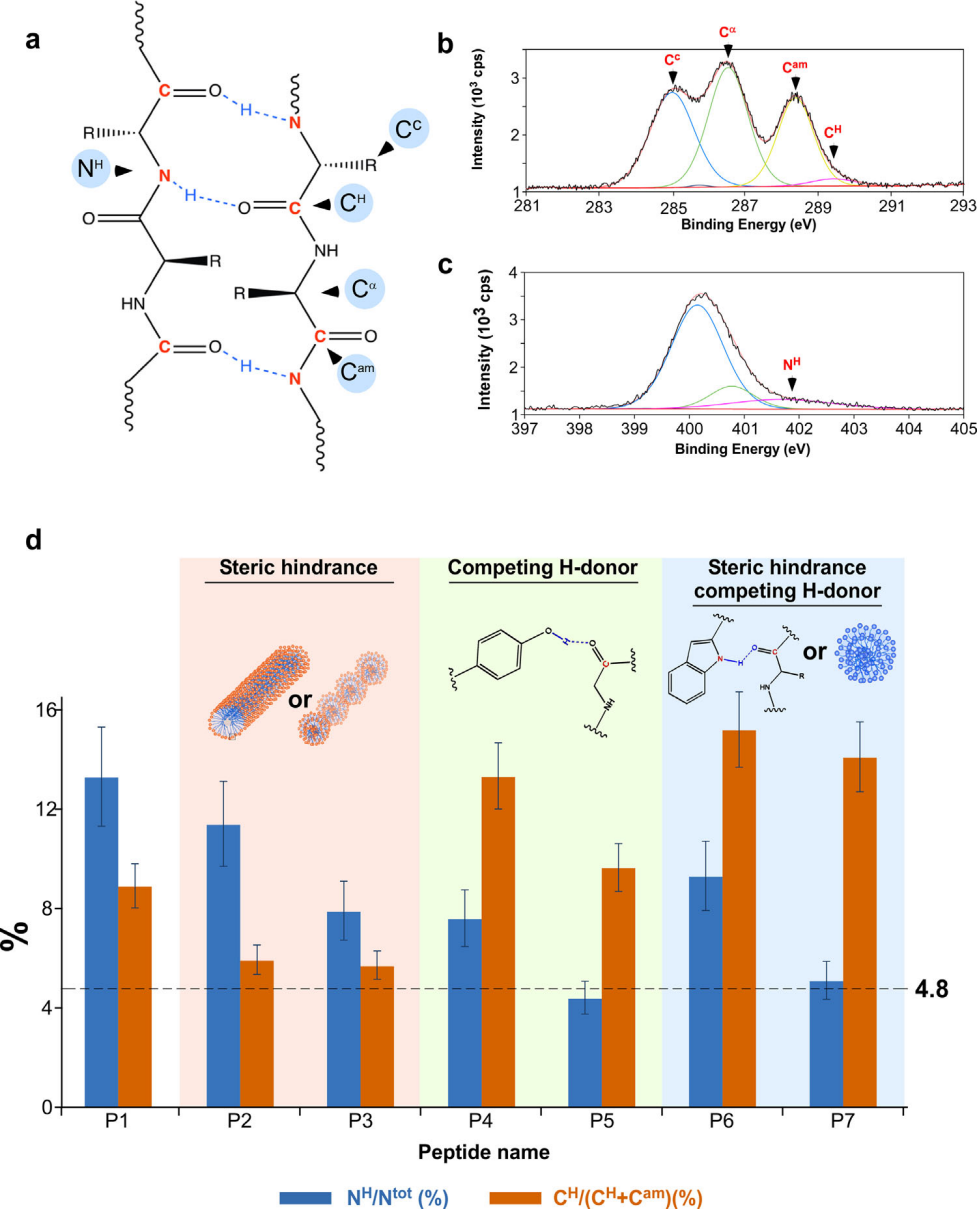
Analysis of H-bonded network formation using X-ray photoelectron spectroscopy. **a** Schematic representation of H-bond formation between the carbonyl of the peptide backbone in one β-strand and the NH of the peptide backbone in another β-strand. **b** Carbon XPS spectrum of P1 showing its different oxidation states. Most interestingly, fingerprints of the H-bond formation can be extracted. **c** Nitrogen XPS spectrum of P1, showing different oxidation states, viz., the bare NH in an amide/peptide backbone and that of NH involved in H-bond formation. **d** Histogram showing the percentage of H-bond formation as expressed by the N 1 s spectrum and by the C^am^ signal, evaluated for the entire set of polymers. Error bars STDV.

The general scheme of H-bond formation in β-sheets, involving a backbone carbonyl and amide nitrogen, is shown in Fig. 3a. The electron density around the O-H bond is expected to increase at expense of both neighboring sites: the nitrogen and the carbon. Accordingly, deconvolution of the carbon spectra yields four peaks. The major peaks, labeled C^C^, C^α^, and C^am^, correspond, respectively to CH_2_/CH_3_, α-carbon, and the amide carbons. The fourth component, C^H^, is a shoulder to C^am^ and is associated (besides the carboxylic end groups of the peptides) with amide carbonyl groups that form an H-bond, e.g., with the NH of a neighboring peptide chain (present in β- strand/ β- sheet)^57^. A representative XPS spectrum for P1 is shown in Fig. 3b. The measured chemical shifts, in reference to C^C^, are 1.54, 3.38, and 4.43 eV for C^α^, C^am^, and C^H^, respectively, with ~1 eV attributed to the H-bond effect on C^am^.

Similarly, the deconvolution of the nitrogen spectra reveals a shoulder at the high binding energy side, attributed (besides end groups) to nitrogen atoms involved in the formation of H-bonds (N^H^) with a carbonyl oxygen of an amide in a neighboring biopolymer backbone. A representative nitrogen spectrum for P1 is shown in Fig. 3c, with a chemical shift of ~1.5 eV, which is higher than that of C^am^, as may be expected intuitively.

Our analysis shows that although the 10% substitution of G for F, Y, or W consistently results in decreased N^H^/N^tot^, it does not necessarily affect the C^H^/(C^H^ + C^am^) ratio (Supplementary Table 2, Supplementary Note 2, Supplementary Fig. S6 and Fig. 3d). A follow-up on the composition is given by the C^H^/ C^am^ and N^H^/N^tot^ ratios. This implies that the aromatic acid side chains can either hinder the H-bond formation between the backbone atoms, and/or provide an alternative H-bond donor (OH in the case of tyrosine, NH in the case of tryptophan). The 10% G-to-F substitution (P2) leads to a decrease in N^H^/N^tot^, indicating the phenylalanine ring’s steric hindrance of H-bond formation, which most likely yields a weaker H-bond. The existence of weaker H-bonds in P2 (10% G-to- F), compared to P1, is hinted (close to the experimental error level) by a decrease in ΔΕ_Β_(N) (Supplementary Table S2, column 4). The 10% G-to-Y substitution (P4) resulted in an up to twofold reduction in N^H^/N^tot^, concomitant with an increase in the C^H^/C^am^ ratio, where 4.8% of the fraction was attributed to the C and N terminal groups. This system demonstrates that, in addition to the steric hindrance created by the aromatic ring of the tyrosine, a chemically active, -OH group (of tyrosine) competes with the backbone NH, providing an alternative donor for H-bond formation with the carbonyl oxygen. The latter argument is supported by an increase in C^H^/C^am^. However, note that we observed a rise in Δ*Ε*_Β_ (Ν) in the 10% G-to-Y variant (in comparison to P1), implying the formation of a stronger H-bond between C^am^ and N^H^. Taken together, these results support the view that the 10% G-to-Y substitution accounts for the increase in the strength of the H-bond between the backbone atoms, concomitantly providing an additional H-bond donor (OH). Similarly, the 10% G-to-W substitution resulted in a decrease in N^H^/N^tot^ and an increase in C^H^/C^am^. This suggests that C^H^ forms an additional H-bond with the NH of the tryptophan indole rings.

At higher concentrations of the added side-groups, F, Y, and W, and when they are distributed along the polymer (and not only next to its end groups), a qualitative difference with regard to steric effect should be considered. Taken together, our results show that a 30% G-toY/W substitution results in the formation of fewer, yet stronger, backbone H-bonds, as manifested by the increase in Δ*Ε*_Β_(N) (Supplementary Table S2 and Supplementary Note 2). We can therefore conclude that the presence of any of the three hydrophobic amino acids, phenylalanine, tyrosine, or tryptophan, introduces steric hindrance, whereas Y and W provide additional H-bond donors. Our XPS results clearly show that the extent of H-bond reduction greatly depends on the type of aromatic acid and its respective extent of incorporation into the sequence. These results are well supported by the FT-IR data, which show an increase in amyloid structure/decrease in native structure.

We next assessed the influence of G to aromatic amino acid substitutions on the structural characteristics of amyloidogenic assemblies. To this end, we performed electron diffraction analysis^58^ on peptide assemblies (Supplementary Fig. S7). The atomic structures of the P1, P2, P3, P6, and P7 assemblies show the cross β-spine characteristics of amyloids. This analysis revealed that G to F substitution, in P2 and P3, affected interstrain distances, namely distances between two peptides forming a single β-strain (~4.7 Å (for P1) to ~4.9 Å (for P2 and P3), but exhibited identical intersheet distances of ~10.8 Å. This result indicates the orientation of F residues at the interface of peptide assemblies, which is reflected in increased interstrain distances (Supplementary Fig. S8). A slight variation in results has been observed for G to Y and for G to W substitutions, in P4, P5, P6, and P7; the interstrain distances have been increased for P4 and P5 (from ~4.7 to ~4.8 Å) and decreased for P6 and P7 (from ~4.7 to ~4.6 Å), whereas intersheet distances remained unchanged (~10.9 Å). Thus, we can conclude that in all the cases of P2-P7 peptide self-assembly, the hydrophobic amino acids F/Y/W in the peptide sequence localize at the fibril interface upon self-assembly (Supplementary Fig. S8).

**Fig. 4:**
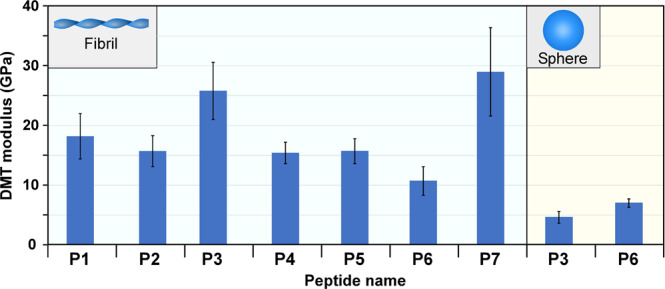
Nanomechanics of supramolecular peptide assemblies. Relationship between the DMT modulus (GPa) and the morphology of the self-assembled P1–P7 peptides. Error bars STDV.

To further understand and to correlate the formation of certain morphologies, H-bonds, and the material properties of the structures, we analyzed the mechanical properties of peptide fibrils, which are summarized in Fig. 4. Overall, two fibrillar peptide assemblies have shown increased elastic modulus values, P3 and P7 (26 and 29 GPa, respectively). Interestingly, the spherical assemblies of the same peptides displayed relatively low modulus values of <5 GPa. This observation highlights the role of the continuous H-bonded network in the formation of fibrils with high stiffness values, whereas the presence of aromatic amino acids contributes to improved stiffness, independently of its localization in the fibril structure.

A number of conclusions can be drawn based on our combined set of experimental results. First, the samples consisting of only two substituted side-groups (at two termini) per peptide backbone were designed to interfere with the early stage of monomers assembly. This process involves relatively strong interactions between the COOH and NH_3_ end-groups and, therefore, the presence of neighboring side-groups is not expected, except for extreme cases, to introduce significant competition during this stage of assembly. Accordingly, the incubation time in all cases of 10% substitution (P2, P4, P6) is nearly unaffected. Furthermore, the presence of these side-groups dictates spatial orientation, influencing the later stages of assembly. An interesting example is provided by the increased rate of elongation for P2 and, even more for P3. The substituted side-groups, aromatic residues, within these complexes are forced sterically to be oriented *out of* plane and, thus, assembly *within* the plane becomes more efficient, simply by entropy considerations.

A second factor affecting the assembly kinetics should be noted here: the strength of molecule-solvent interactions. It is well known that closed (spherical) nanostructures of peptide clusters are typically formed at intermediate stages of growth^59^. As long as their stability is low, the efficiency of fibrillar growth can take over. However, P3 and P6 manifest a more challenging competition in that sense. In contrast to the Y side-group (P4, P5), for which no affinity to DMSO is noted, the F group presents significant attractive interactions with the DMSO solvent and, even more, is the strength of W (amino acid)-DMSO interactions by the means of solvation. As a result, in spite of the increased elongation rate of P3 (explained above to arise from orientation ordering of early peptide complexes), we found in this case relatively large incubation periods and, also, significant amounts of spherical clusters, both results indicating the increased role of phenyl-DMSO interactions. The W side-groups present even stronger interactions, which results in *no formation* of fibrils for P6. Furthermore, we probed the aggregation-prone behavior of the peptides in aqueous environment. Our observation (Supplementary Fig. S9), indicates that all peptides were found to be unstable in aqueous environment (<1% water) and tend to fibrillate even in the presence of water vapors (~60% humidity) due to the hydrophobic nature of the core sequence.

Intriguingly, with a sufficiently large number of W side-groups, in P7, the incubation time is extremely long and, yet, fibrils are eventually formed. They exhibit poorer inner order, indicating that the fibrillar growth evolves via mutual interactions between the side-groups, thus opening alternative paths of growth. Remarkably, the latter factor, inter-peptide interactions provided by the side groups, is best exemplified by the Y side-groups, where the OH group forms very efficient H-bonds between early peptide complexes. In contrast to P2 and P3, the fibrillar growth for P4 and P5 is more likely to evolve along and via the side groups. The end result is a fast elongation and improved fiber length and flexibility, enabled by the OH side groups.

In summary, herein we described a general approach for which the controlled intervention into molecular interactions, via tailoring side groups to the individual peptide backbones, enables modulation of the H-bonded network and the fibrillation pathway in amyloids. Such intervention directly reflected as changes in nanomechanical properties of fibrillar assemblies. Our combined set of experimental results included kinetics studies, AFM, XPS, FT-IR, and electron diffraction. We have identified at least three competing driving forces that dominate the fibril formation process. The first, geometrical constraint or steric disturbances, found to play a significant role in early peptide-peptide interactions and formation of nucleation sites. Interestingly, such constraints do not necessarily interfere with 2D packing (formation of β-sheet structures) but introduce the changes into the 3D assembly pathways. The second driving force arises from H-bond formation modulated by the amyloidogenic motif sequences, especially by the side groups, thus opening new pathways for assembly. The third driving force involves the side-group affinity to solvent molecules, demonstrating in some cases severe competition with the fibrillar growth. Moreover, the nanomechanics of the fibrillar assemblies modulated by the localization of the side groups either on the fibril interface or in the fibril core leads to the formation of either stiffer or more compliant structures, respectively. This triple-parameter space is limitedly, yet effectively captured by the present set of peptides, thus allowing an insightful qualitative understanding of the interplay between molecular-level forces and resultant macroscopic shapes and mechanical properties.

## Methods

### P1-P7 peptides

The lyophilized peptides (Hylabs, Israel) with the following sequences were used in our studies: GAGAGSGAGAGSGAGAGSGAG (P1), FAGAGSGAGAGSGAGAGSGAF (P2), FAGAFSGAGAFSGAFAFSGAF (P3), YAGAGSGAGAGSGAGAGSGAY (P4), YAGAYSGAGAYSGAYAYSGAY (P5), WAGAGSGAGAGSGAGAGSGAW (P6), and WAGAWSGAGAWSGAWAWSGAW (P7). The folding of peptide monomers was predicted by using PEP FOLD 3 software (https://bioserv.rpbs.univ-paris-diderot.fr/services/PEP-FOLD/).

PEP-FOLD is a de novo approach aimed at predicting peptide structures from amino acid sequences. This method, based on structural alphabet SA letters to describe the conformations of four consecutive residues, couples the predicted series of SA letters to a greedy algorithm and a coarse-grained force field^60^.

### Preparation of peptide self-assemblies

The lyophilized peptides were dissolved in DMSO and then aggregated and kept at 65 °C for 11 days. The aggregates were then analyzed using Fourier transform infra-red spectroscopy (FT-IR), atomic force microscopy (AFM), and X-ray photoelectron spectroscopy (XPS).

### Chemical kinetic measurements based on the ThT assay

The lyophilized peptides were dissolved in DMSO and sonicated in a water bath at 37 °C for 10 min. Undissolved peptides were removed by sample centrifugation at 14,000 rpm for 10 min. For the kinetics assay, 0.3 µM of the obtained (see the Methods section “peptide sample preparation for aggregation”) peptides were mixed with 20 µM of ThT and placed in a 96-well plate (Greiner Bio-One GmbH, Germany) at 65 °C. The process of the self-assembly of peptides was monitored using a Clariostar plate reader (BMG Labtech, Germany). The fluorescence excitation and emission wavelength used for monitoring peptide aggregation were 440 and 490 nm, respectively. The reaction was stopped once the saturation phase of kinetics was reached.

The stability of the peptides in aqueous environment was probed by titrating DDW into peptide-containing DMSO solution and by incubation of the peptide powder under humid (~50–60%) conditions. In the presence of water (<1%) peptides, which pre-dissolved in DMSO, precipitated. Under humid conditions, of ~50–60% humidity, P1-P7 were stable for few months (up to 4 months) as a powder, however, formed fibrillar aggregates after 4 months-12 months storage.

### Fourier transform infra-red spectroscopy (FT-IR) analysis

A 50 µl sample containing treated (aggregated) or untreated (non-aggregated) peptides was placed on FT-IR cards (International Crystal Laboratories, New Jersey) and was allowed to dry under vacuum overnight. The absorbance of the peptides was monitored on a Nicolet 6700 single-beam FT-IR (Thermo Electron Corporation, Massachusetts) between 400 and 4000 cm^−1^ (wavenumber), using the following machine settings: 32 scans/sample, Happ- Genzel Apodization, and a sample spacing of 1.0 cm^−1^. The obtained spectra were subtracted from the DMSO spectra. The second derivative of the spectra and the area under the peaks was determined using Origin (Origin Lab, Northampton, MA).

### Atomic force microscopy (AFM) analysis

An aggregated sample drop (5 µl) was placed on freshly cleaved mica and incubated at RT for 5 min. The excess contacting a filter paper to the edge of the drop. The mica was washed 3-4 times with water and subsequently dried with a nitrogen stream. The sample was imaged on an AFM (JPK Nano wizard 4 AFM, Germany) using AC240 or AC160 cantilevers in tapping mode. The images were processed with JPK data processing software. The mechanical properties of the peptides were measured using “peak force QNM” on a Multimode AFM (Bruker). AC160 (Olympus) or RTESP probes (Bruker) were used with calibrated spring constants between 40 and 50 N/m. The tip area function was calibrated on a sample of HOPG with a modulus of 18 GPa. Deformation depths were kept to about 1 nm. Finally, the built-in software computed the elastic modulus by fitting the force vs. deformation curves to a DMT model.

### X-ray photoelectron spectroscopy (XPS) analysis

A 10 µl-treated (aggregated) sample was spread on a 1-in. silicon wafer and dried overnight in a vacuum. Thus, large areas of uniform thin films were formed (an average thickness of a few nm, disregarding possible holes and discontinuities). XPS measurements were performed in a Kratos AXIS-Ultra DLD spectrometer, using a monochromatic Al kα source at low power, 15−75 W, and with detection-pass energies of 20−80 eV. The pressure in the analysis chamber was kept below 1 × 10^−9^ torr. The energy scale was corrected for surface charging effects by setting the C 1s peak to 285.0 eV, used as a convenient reference with no attempt to get an absolute scaling.

In this respect, it should be stressed that our quantitative analysis of H-bonds is based on the fine details in the line shape of selected signals, which makes it extremely sensitive to *differential* charging, an inherent artifact in the XPS of poorly conducting specimens. Hence, a challenging requirement for minimizing charging-related spectral distortions was encountered here. We used repeated scans under various charging conditions, controlled by the electron flood gun parameters and the X-ray source power, to address this issue. Differentiation of artifacts from the “real” chemical information was also improved by comparing the line shapes of different signals, based on the assumption that, for any given location in the sample, the charging line shifts would be the same for all signals. This approximation applies, in particular, to the line shapes of C^α^ and C^am^, for which we expect to obtain identical spectral distortions, thus enabling an improved derivation of the chemical information hidden in the former. Exceptions beyond this approximation, e.g., ones involving atomic-scale charge redistribution^61^, were found to be of negligible relevance in the systems studied here. Combined with the repeated scans, work-function measurements^62^ at reduced source power, <0.3 W, were applied, in order to better follow the time evolution of the surface potential.

By performing repeated scans, we could further evaluate the effects of beam-induced damage, an issue of general concern upon exposure of organic compounds to X-rays^63^. Such effects were found to be rather small with the reduced beam fluxes used. Minor corrections were applied when needed by inspecting the time evolution of spectral signals and eventually extrapolating towards zero exposure data. The reader is referred to an XPS study of α-helix structures, in which similar signatures of the molecules and H-bonds formation could be resolved spectrally, however encountering significantly faster damage evolution under the X-ray beam^64^.

## Supplementary information

Supplementary Information

## Data Availability

The datasets generated and analyzed during the current study are available from the corresponding author on reasonable request.
