# Supplementary material for: Protein nanofibril design via manipulation of hydrogen bonds
Source: Commun Chem. 2021 May 11;4:62. doi: 10.1038/s42004-021-00494-2 (PMC9814780; doi:10.1038/s42004-021-00494-2)
Supplement: Supplementary file 1 — Supplementary Information [file 42004_2021_494_MOESM1_ESM.pdf]

Protein nanofibril design via manipulation of hydrogen bonds

Nidhi Aggarwal[1#], Dror Eliaz[1#], Hagai Cohen[2], Irit Rosenhek-Goldian[2], Sidney R. Cohen2, Anna Kozell[1], Thomas O. Mason[1], and Ulyana Shimanovich[1]*

[1]Department of Molecular Chemistry and Materials Science, Weizmann Institute of Science, 76100 Rehovot, Israel
[2]Department of Chemical Research Support, Weizmann Institute of Science, 76100 Rehovot, Israel

[#] These authors contributed equally to this work.
* to whom correspondence should be addressed: ulyana.shimanovich@weizmann.ac.il.

Keywords: amyloid, protein nanofibrils, hydrogen bonds, protein materials, self-assembly

Supplementary information

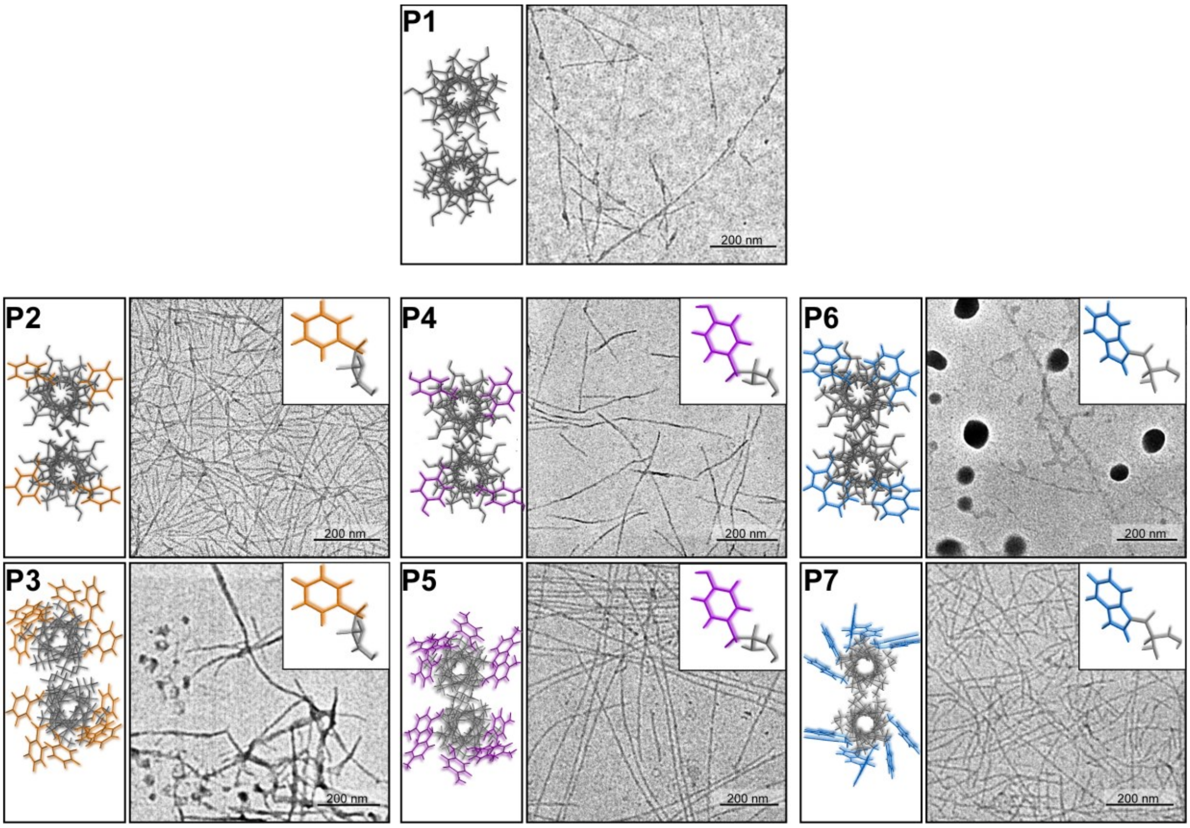

Supplementary Figure 1. Electron microscopy analysis of P1-P7 peptide self-assemblies: (P1) in which G is substituted with F in P2, Y in P4, or W in P6 aromatic amino acids (10% substitution) and when the fraction of F (P3), Y (P5), and W (P7) was increased to 30%. The chemical structure of aromatic residue is shown as an insert in the right top corner. The peptide-peptide interaction was predicted using PEP-FOLD3 (shown in ribbon and stick formats, left panel) [1]. The morphology of the self-assembled peptides, determined by TEM (the middle panel), indicates the existence of polymorphism. The scale bars for the TEM images are 200nm.

a

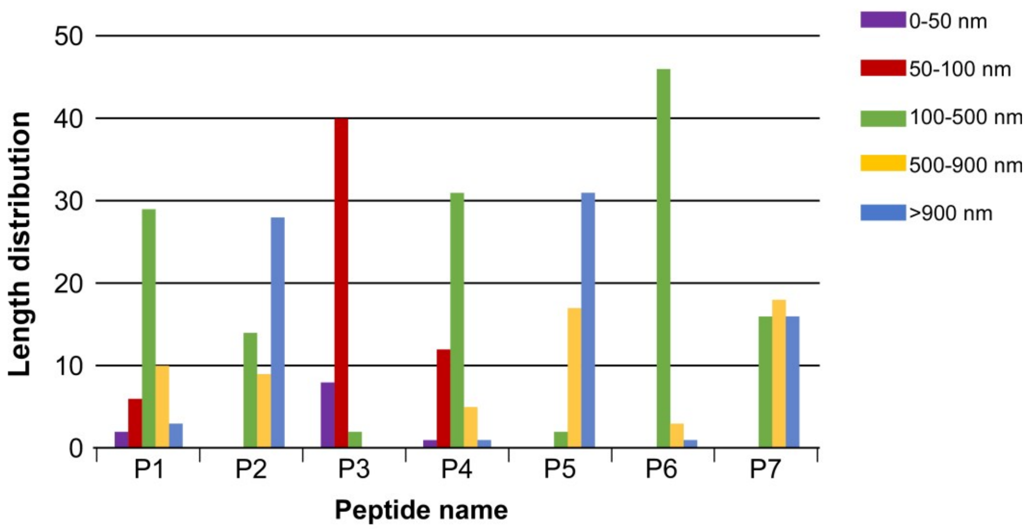

b

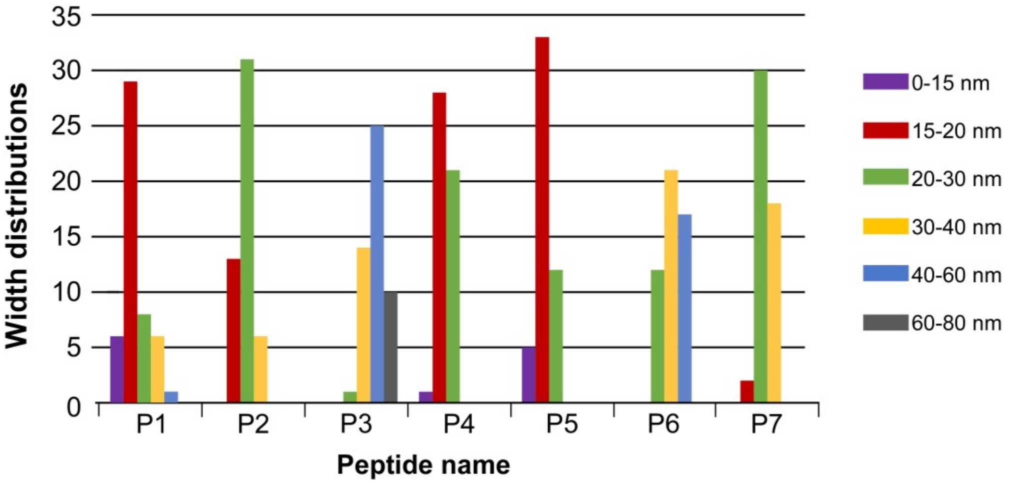

Supplementary Figure 2. Size, length and width distribution for P1-P7 fibrillar peptides. Histograms showing the **a** length distribution and **b** width distribution of the fibrillar nanostructures obtained upon the self-assembly of the P1-P7 peptides. The length and width distributions were calculated from AFM images and shown on *the right hand side* of each histogram.

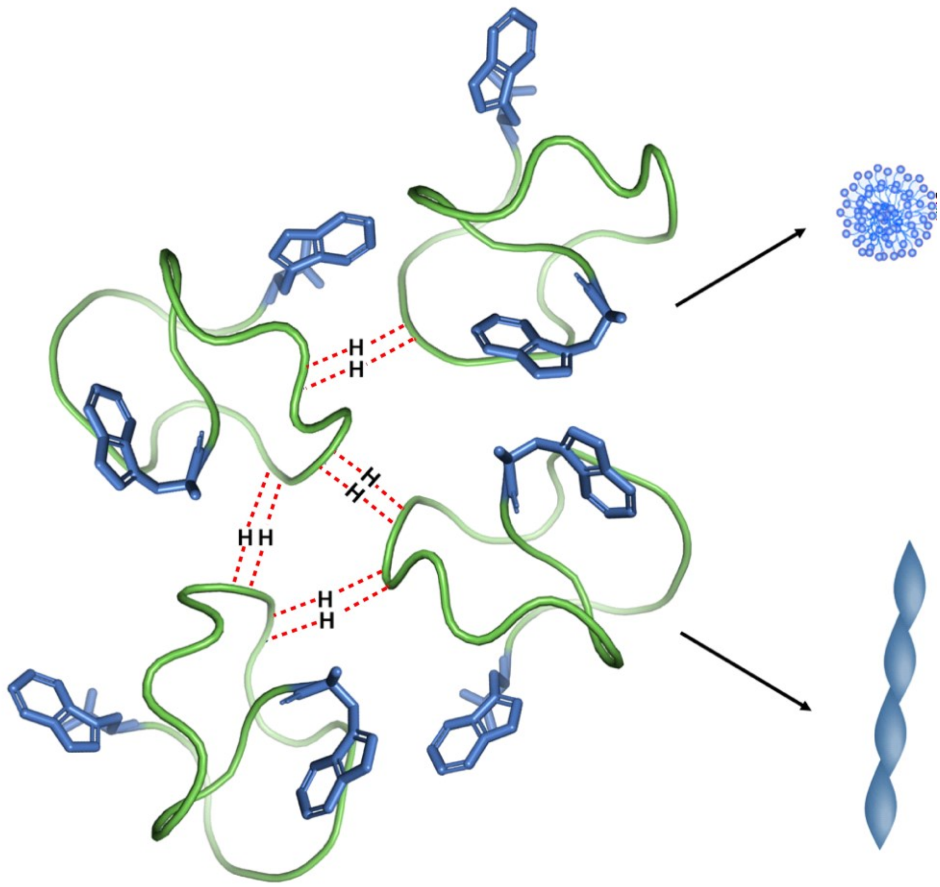

Supplementary Figure 3. Schematics depicting two types of H-bonded interactions in fibrillar peptide assemblies. Schematic representation of the orientation of tryptophan residues upon the self-assembly of peptide P6 (G-to-W substitution), which limits the growth and hence, the formation of spheres and fibrils. In both self-assembly cases, fibrillar and spherical, the hydrophobic residues (tryptophan) are oriented toward the outer environment. The H-bonds between peptide backbones are marked with red dashed lines.

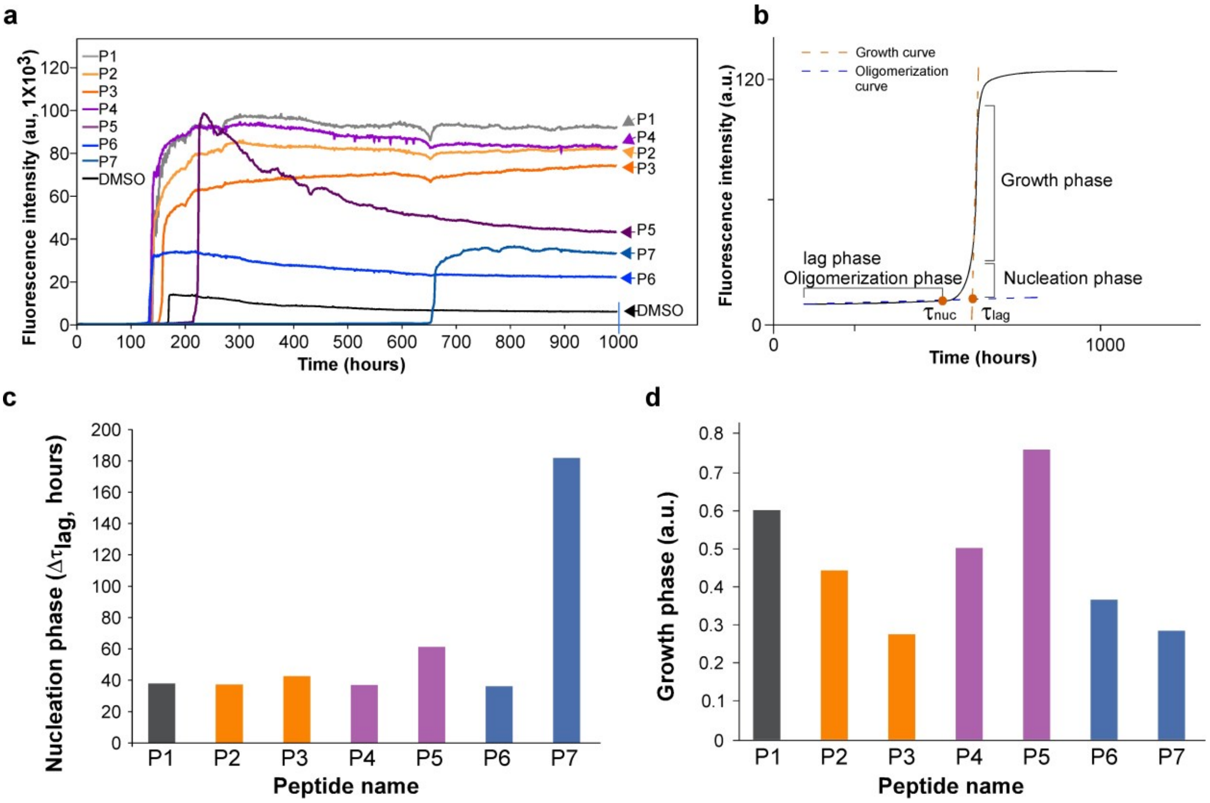

Supplementary Figure 4. Analysis of chemical kinetics for P1-P7 fibrillar peptide self-assembly process. a The kinetics of the fibrillar aggregation of P1-P7 peptides as determined by the increase in red-shifted ThT fluorescence upon binding to self-assembling peptides, with measured excitation and emission maxima at 440 and 490 nm, respectively. **b** Kinetics profile of amyloid protein fibrillation phenomenon with indicative key molecular events, namely nucleation, oligomerization and growth phases. **c** Analysis of nucleation phase for P1-P7 peptides. **d** Analysis of growth phase for P1-P7 peptides.

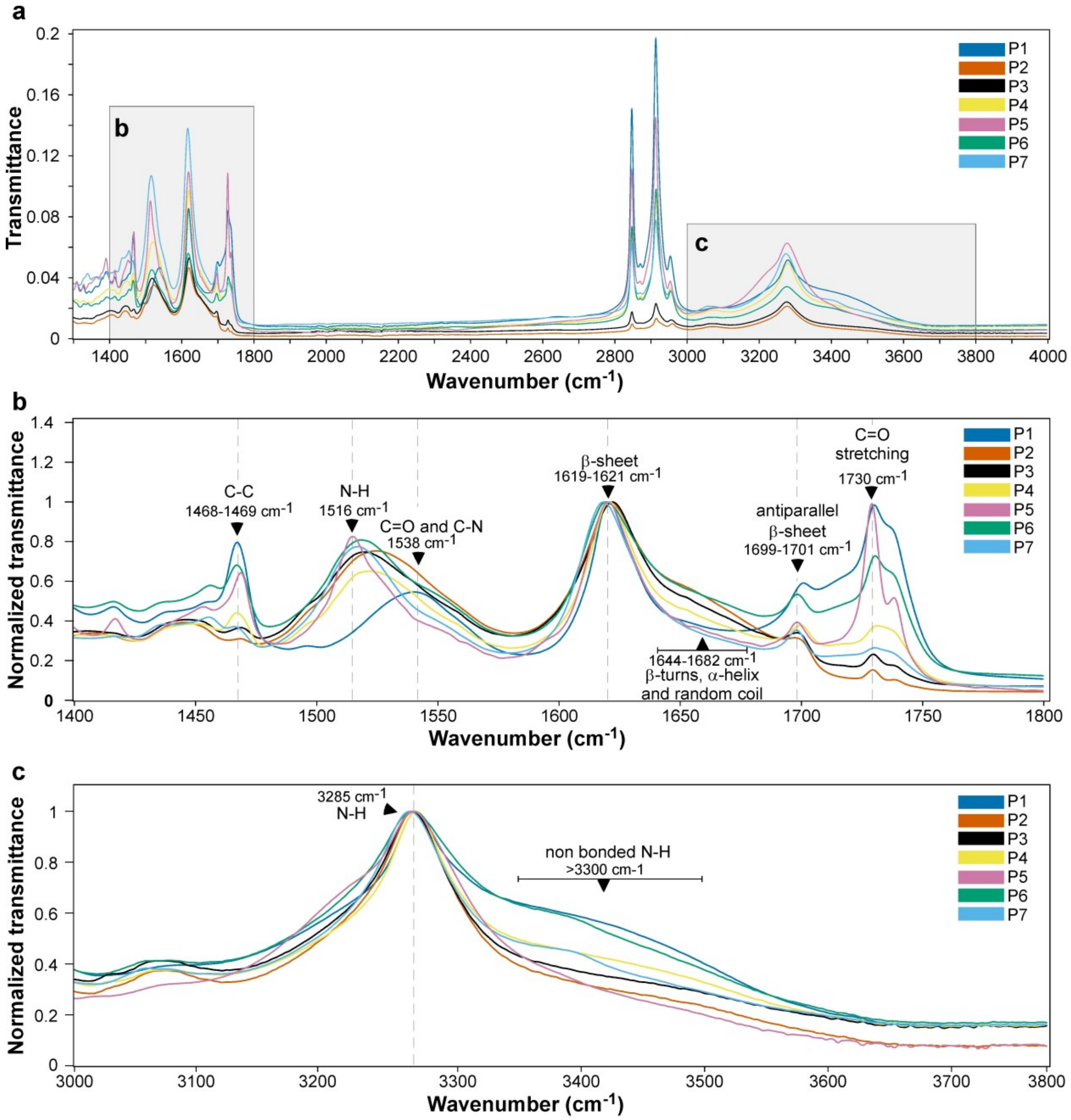

Supplementary Figure 5. **Secondary structure analysis of self-assembled P1-P7 peptides by Fourier transform infrared spectroscopy. a** Full Fourier transform infrared spectroscopy (FT-IR) spectra of self-assembled peptides (P1-P7) showing the existence of variations in the secondary structures of peptides' assemblies (intermolecular β-sheet (1610−1625 cm^{-1}), native β-sheet (1625−1635 cm^{-1}), random coil)/α-helix (1635−1665 cm^{-1}), β-turn (1665−1690 cm^{-1}), and anti-parallel amyloid β-sheet (1690−1705 cm^{-1})). The assignment of vibration bands, stretching and bending, are shown as labels in spectra. **b** Enlarged FT-IR spectra of amide I and amide II regions with assigned chemical bonds. **c** Enlarged FT-IR spectrum of the amide A region with assigned relevant chemical bonds.

Supplementary Note 1: The XPS-derived composition, presented as atomic concentration ratios, is summarized in Supplementary Table 1. A small deviation from the theoretical predictions was mainly seen in the amounts of oxygen and non-oxidized carbon (C^C, e.g., C-C, CH), which we attribute to adventitious hydrocarbon and water molecules adsorbed on the peptides layers. However, these foreign moieties do not interfere with our quantitative analysis of H-bonds, since they rely on two signals that are well resolved from those of the foreign moieties; the N 1s line and the highly oxidized C^{am} component of the amide groups, both of which agree very well with the theoretically predicted values (**Supplementary Table 1**).

	N/C^{am}		C^{α}/C^{am}		C^C/C^{am}		O/C^{am}	
	**Theory**	**exp.**	**Theory**	**exp.**	**Theory**	**exp.**	**Theory**	**exp.**
**P1**	1	0.96	1.142	1.315	0.333	1.215	1.19	1.5
**P2**	1	0.99	1.142	1.235	1	1.8	1.19	1.52
**P3**	1	1.015	1.142	1.54	1	3.21	1.19	1.195
**P4**	1	1.05	1.238	1.49	0.904	2.17	1.286	1.98
**P5**	1	1.015	1.428	1.935	2.047	3.07	1.476	2.13
**P6**	1.095	1.035	1.142	1.56	1.19	2.325	1.19	2.08
**P7**	1.285	1.245	1.142	1.825	2.904	4.185	1.19	2.345

Supplementary Table 1. **Summary of binding energies for amide carbon C^{am} measured by X-ray photoelectron spectroscopy.** XPS-derived atomic concentrations, normalized to the signal of amide carbon, C^{am}. For technical reasons, we include in C^{am} both the actual amide carbon and the COOH end groups, where theoretically the latter is about 4.8% in magnitude. The presented oxygen amounts correspond to values after the native SiO_2 contribution by the Si substrate was subtracted. The experimental relative error is up to 10% for N and C signals and ≤15% for the oxygen.

Supplementary Note 2: Our quantitative XPS analysis of H-bonds relies mainly on the signal intensities, as derived from the area under the curve of related components and normalized to corresponding total intensities (perturbed plus unperturbed): namely, N^H/N^{tot} and $C^H/(C^H+C^{am})$. The normalized values are indicative of the percentage of hydrogen bonds formed in each system, provided that the bare contribution to these spectral shoulders by end groups of the chain, COOH and NH_3^+, is taken into account. Additionally, chemical shifts are evaluated. They should correlate with the magnitude of charge transfer, but experimentally, their evaluation is not as accurate. Results are summarized for the entire set of peptides in **Supplementary Table 2** (see also **Figure 3d**).

	N^H/N^{tot}	C^H/C^{am}	S/N^{tot}	$\Delta E_B(N)$	$\Delta E_B(C)$
**P1**	13.2	11	5	1.53	1.04
**P2**	11.6	8.2	2	1.25	1.1
**P3**	7.9	6	< 1.6	1.04	1.06
**P4**	7.2	9.4	< 0.5	1.73	1.04
**P5**	4.4	9.6	< 1.5	1.82	1.04
**P6**	9.3	15.2	< 1	1.6	1
**P7**	5.1	14	< 1	1.69	1.1

Supplementary Table 2. **Evaluation of the H-bond-related signals by X-ray photoelectron spectroscopy.** The two inspected spectral tails are normalized (values are given in %) to the total signal of each corresponding line. The presence of residual sulfur is expressed by the S/N^{tot} ratio (given in %). H-bond related chemical shifts, $\Delta E_B(N)$ and $\Delta E_B(C)$, given in eV, are shown in the right columns. The relative error in intensity ratios is up to 20% and the error in ΔE_B is ≤0.2 eV.

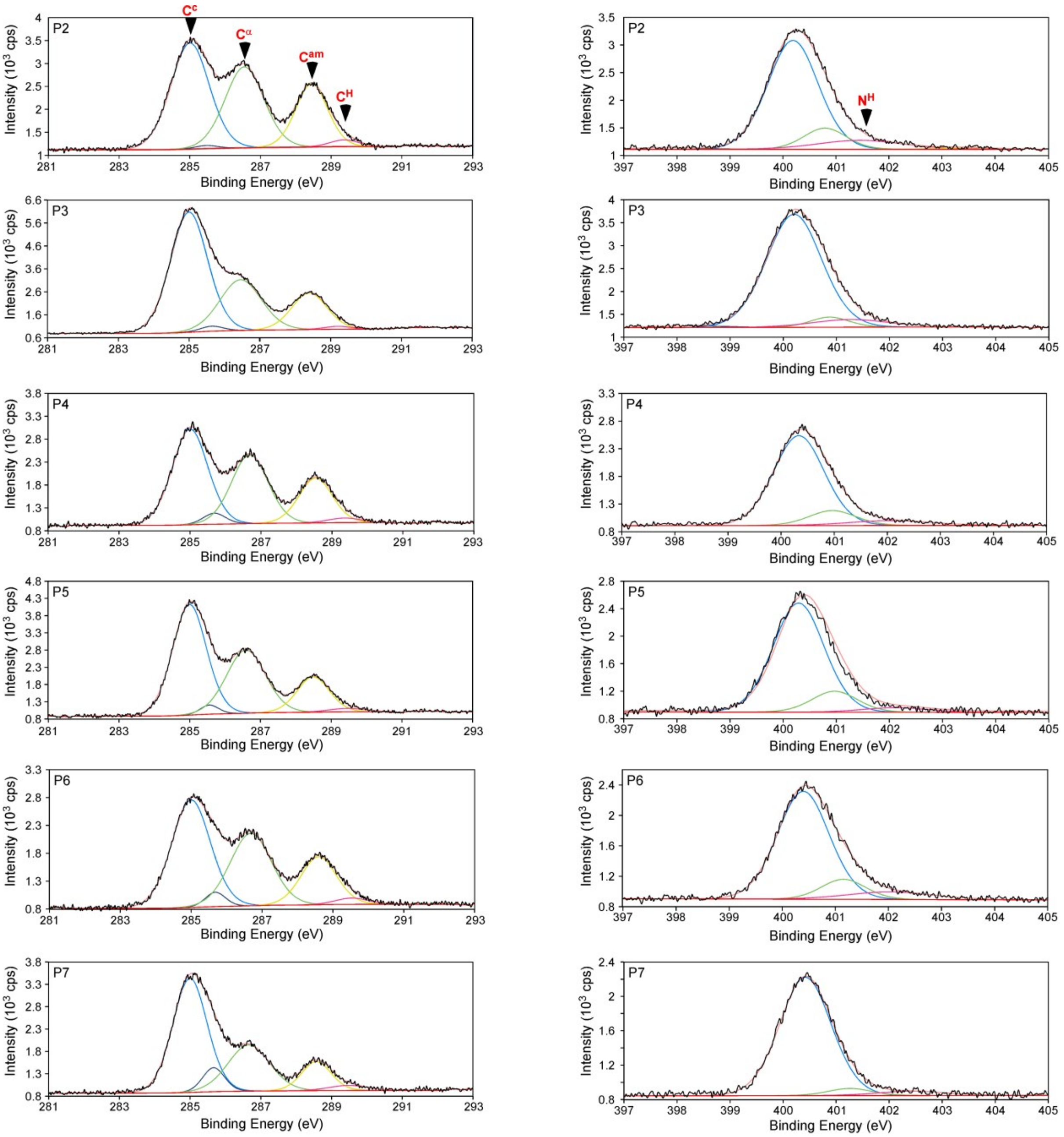

Supplementary Figure 6. **X-ray photoelectron spectroscopy spectra of carbon C 1s and nitrogen N 1s in self-assembled peptides**. XPS spectra of self-assembled peptides showing the curve fitting results and, in particular, the indications for different percentages of H-bonded backbone atoms. The H-bond related components, C^H and N^H, are indicated respectively in the top panels of the left and right columns.

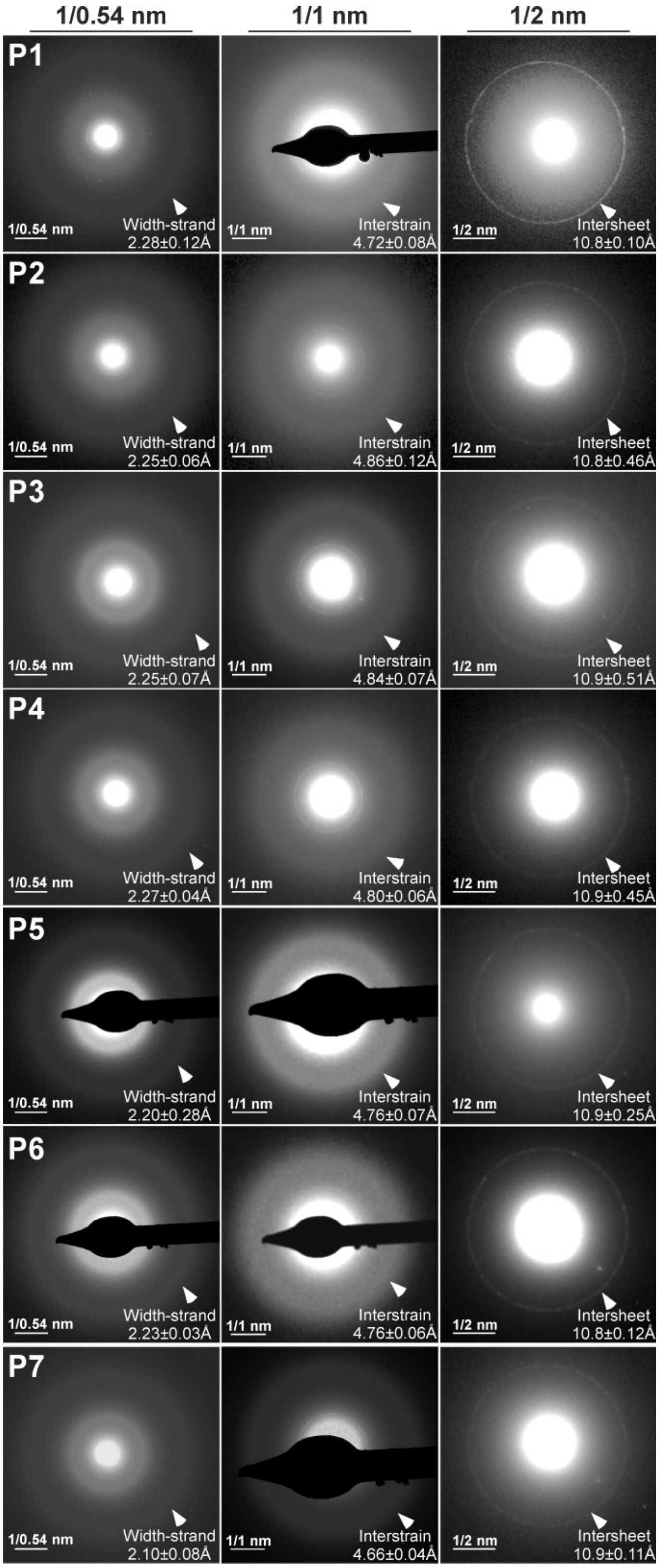

Supplementary Figure 7. **Electron micrographs showing the electron diffraction pattern of self-assembled peptides P1-P7.** The measured values of width strand, interstrain, and intersheet distances for each peptide assembly are shown as an insert.

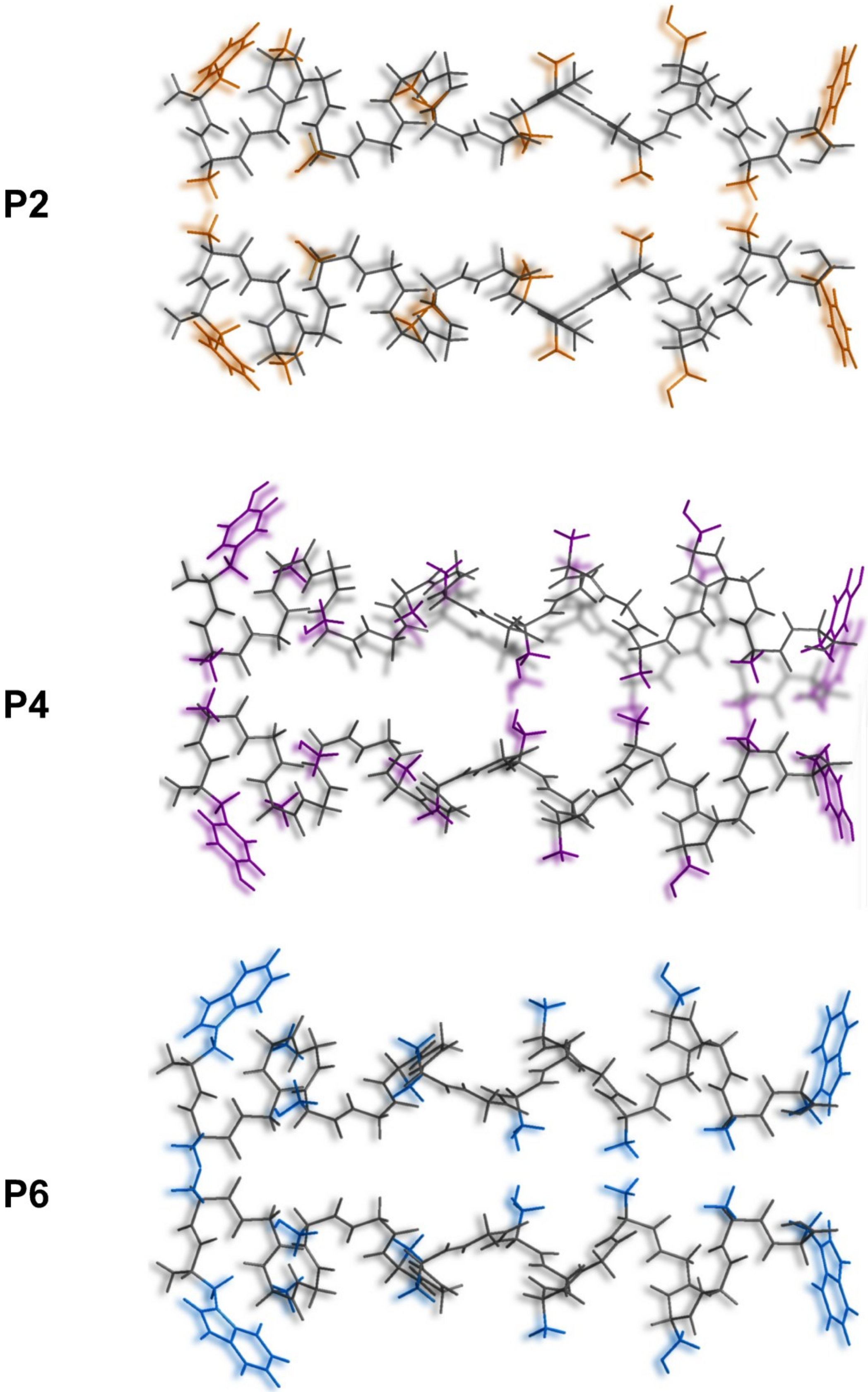

Supplementary Figure 8. **Schematic representation of the interactions between two peptide molecules.** In all the cases the hydrophobic residues face an external part.

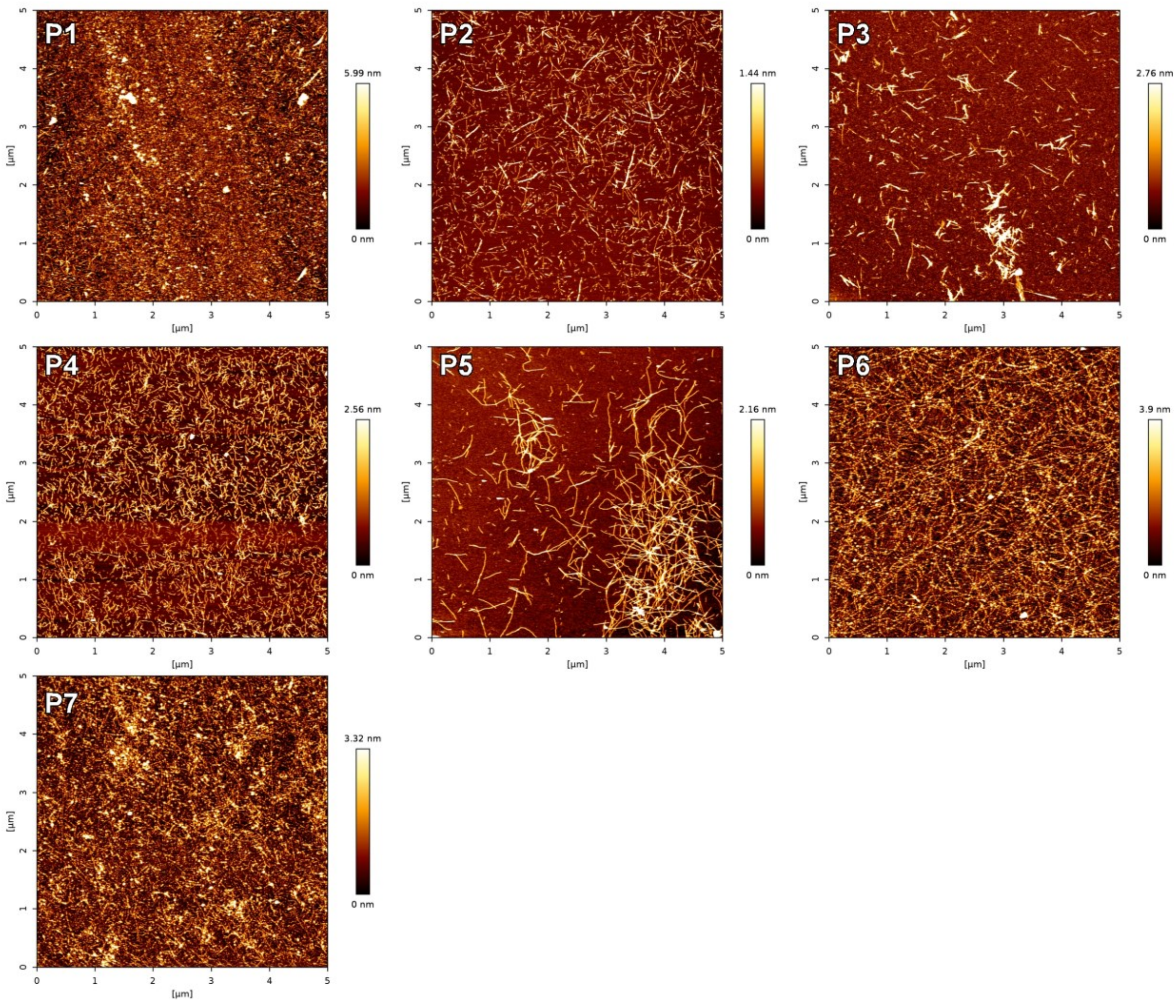

Supplementary Figure 9. **Atomic force microscopy images of P1-P7 fibrillar peptide assemblies formed in aqueous environment.** The peptides (powder) kept at the 4°C up to 1 year, at ~50% of humidity. Each peptide was re-dissolved in DMSO to final concentration of 1 mg/ml.

Supplementary References

1. Shen, Y., Maupetit, J., Derreumaux, P. & Tufféry, P. Improved PEP-FOLD Approach for Peptide and Miniprotein Structure Prediction. *J. Chem. Theory Comput.* **10**, 4745–4758 (2014).
